# Sociodemographic Factors and Trends in Bronchiolitis-Related Emergency Department Visit and Hospitalization Rates

**DOI:** 10.1001/jamanetworkopen.2024.8976

**Published:** 2024-04-29

**Authors:** Sanjay Mahant, Cornelia M. Borkhoff, Patricia C. Parkin, Haris Imsirovic, Meltem Tuna, Colin Macarthur, Teresa To, Peter J. Gill

**Affiliations:** 1 Child Health Evaluative Sciences, The Hospital for Sick Children Research Institute, Toronto, Ontario, Canada; 2 Division of Pediatric Medicine, Department of Pediatrics, University of Toronto, Toronto, Ontario, Canada; 3 Institute of Health Policy, Management, and Evaluation, Dalla Lana School of Public Health, University of Toronto, Toronto, Ontario, Canada; 4 Division of Pediatric Medicine and the Pediatric Outcomes Research Team, The Hospital for Sick Children, Toronto, Ontario, Canada; 5 ICES, Ottawa, Ontario, Canada; 6 The Ottawa Hospital Research Institute, Ottawa, Ontario, Canada

## Abstract

**Importance:**

Bronchiolitis is the most common and most cumulatively expensive condition in pediatric hospital care. Few population-based studies have examined health inequalities in bronchiolitis outcomes over time.

**Objective:**

To examine trends in bronchiolitis-related emergency department (ED) visit and hospitalization rates by sociodemographic factors in a universally funded health care system.

**Design, Setting, and Participants:**

This repeated cross-sectional cohort study was performed from April 1, 2004, to March 31, 2022, using population-based health administrative data from children younger than 2 years in Ontario, Canada.

**Main Outcome and Measures:**

Bronchiolitis ED visit and hospitalization rates per 1000 person-years reported for the equity stratifiers of sex, residence location (rural vs urban), and material resources quintile. Trends in annual rates by equity stratifiers were analyzed using joinpoint regression and estimating the average annual percentage change (AAPC) with 95% CI and the absolute difference in AAPC with 95% CI from April 1, 2004, to March 31, 2020.

**Results:**

Of 2 921 573 children included in the study, 1 422 088 (48.7%) were female and 2 619 139 (89.6%) lived in an urban location. Emergency department visit and hospitalization rates were highest for boys, those with rural residence, and those with least material resources. There were no significant between-group absolute differences in the AAPC in ED visits per 1000 person-years by sex (female vs male; 0.22; 95% CI, −0.92 to 1.35; *P* = .71), residence (rural vs urban; −0.31; 95% CI −1.70 to 1.09; *P* = .67), or material resources (quintile 5 vs 1; −1.17; 95% CI, −2.57 to 0.22; *P* = .10). Similarly, there were no significant between-group absolute differences in the AAPC in hospitalizations per 1000 person-years by sex (female vs male; 0.53; 95% CI, −1.11 to 2.17; *P* = .53), residence (rural vs urban; −0.62; 95% CI, −2.63 to 1.40; *P* = .55), or material resources (quintile 5 vs 1; −0.93; 95% CI −3.80 to 1.93; *P* = .52).

**Conclusions and Relevance:**

In this population-based cohort study of children in a universally funded health care system, inequalities in bronchiolitis ED visit and hospitalization rates did not improve over time.

## Key Points

**Question:**

Have inequalities based on sex, residence location, and material resources in bronchiolitis emergency department visits and hospitalizations improved over time?

**Findings:**

In a population-based cohort study of 2 921 573 children from 2004 to 2022 in Ontario, Canada, bronchiolitis emergency department and hospitalization rates were highest for boys, those with rural residence, and those with lowest material resources. There were no between-group differences in the average annual percentage change in bronchiolitis emergency department visit and hospitalization rates for sex (female vs male), residence (rural vs urban), and material resources (greatest vs least).

**Meaning:**

These findings indicate that inequalities in bronchiolitis emergency department visit and hospitalization rates did not improve over time.

## Teaser

This cohort study of children younger than 2 years in Ontario, Canada examines temporal trends in rates of bronchiolitis-related emergency department visits and hospitalization.

## Introduction

Quantifying and assessing health inequalities in a population is important as health care systems seek to reduce health inequities.^1,2,3^ The World Health Organization defines health inequalities as “differences in health status or the distribution of health determinants between different population groups.”^4^ Health inequalities are assessed by comparing differences in health outcomes among population groups. Health inequalities may be due to biological variations, personal preferences, or an individual’s environment and conditions outside their control. When the differences in health outcomes are avoidable or deemed unfair and unjust, they are termed health inequities.^5^ Equity stratifiers, also called dimensions of inequality, are characteristics chosen to investigate perceived inequalities. The PROGRESS (place of residence, race/ethnicity/culture/language, occupation, gender/sex, religion, education, socioeconomic status, and social capital) framework summarizes the equity stratifiers most frequently used when grouping individuals into population subgroups to measure health inequalities^6^

Bronchiolitis is a common cause of childhood emergency department (ED) visits and hospitalizations and is among the costliest conditions in pediatric hospital care. Health inequalities in bronchiolitis may occur for several reasons.^7,8^ Material hardship, rural residence, and male sex have been associated with greater health care use and poorer outcomes.^6,9,10,11,12,13,14^ Although several studies have examined health inequalities in bronchiolitis outcomes,^15,16,17,18,19^ few studies have examined bronchiolitis ED visit and hospitalization inequalities over time using population-based data.^20^ Such research is important to understand trends in inequality and whether the inequality gap is narrowing or widening. Therefore, the objective of this study was to conduct a population-based cohort study within a universally funded health care system in Ontario, Canada, to examine temporal trends in bronchiolitis-related ED visit and hospitalization rates from 2004 to 2022. Specifically, we examined trends in bronchiolitis outcomes by equity stratifiers: sex, residence location, and material resources.

## Methods

### Study Design

We conducted a population-based cohort study using a repeated cross-sectional study design to examine trends in bronchiolitis-related ED and hospitalization rates by sociodemographic factors in Ontario, Canada. In a similar approach to previous studies,^13,14^ the data sources were provincial health administrative databases housed at ICES (formerly known as the Institute for Clinical Evaluative Sciences), an independent, nonprofit research institute whose legal status under Ontario’s health information privacy law allows it to collect and analyze health data for health system evaluation without patient consent. Data were collected from children younger than 2 years from April 1, 2004, to March 31, 2022. Annual (April 1 to March 31) rates of bronchiolitis ED visits and hospitalizations were determined for the entire study period. The trend analysis, which determined the average annual percentage change (AAPC) in rates and differences in AAPC for the equity stratifiers, focused on April 1, 2004, to March 31, 2020. We did not include the period from April 1, 2020, to March 31, 2022 (the last 2 annual time points), in the trend analysis given the unprecedented reduction in bronchiolitis incidence during the early COVID-19 pandemic period. Research ethics board approval was obtained from The Hospital for Sick Children. The study followed the Strengthening the Reporting of Observational Studies in Epidemiology (STROBE) reporting guideline.^21^

### Study Population

All children younger than 2 years who were residents of Ontario and enrolled in the provincially funded Ontario Health Insurance Plan (OHIP) were included. Data from all children’s and community hospitals in the province were included. Bronchiolitis encounters were identified using *International Statistical Classification of Diseases and Related Problems, Tenth Revision, Canada* (*ICD-10-CA*) discharge diagnosis codes for bronchiolitis, including both respiratory syncytial virus (RSV) and non-RSV causes, and whether bronchiolitis was the primary or secondary discharge diagnosis (eMethods in Supplement 1). Viral pneumonia diagnosis codes (eg, RSV, influenza, and adenovirus pneumonia) were also included because viral pneumonia presents similarly and is managed similarly to bronchiolitis in children younger than 2 years.

### Data Sources

Health administrative databases,^22^ linked using unique encoded identifiers derived from the OHIP number of every resident in Ontario with public health insurance, were used (eMethods in Supplement 1 contains information on the databases). The Registered Persons Database includes demographic information on all Ontarians registered for public health insurance and was used to obtain age, sex, and residence postal code. The National Ambulatory Care Reporting System database was used to obtain ED visit data, the Canadian Institute for Health Information Discharge Abstract Database was used for hospitalization data, and the MOMBABY database was used to capture birth records for inpatient births (the database captures more than 97% of all births in Ontario).^23^

### Patient Characteristics

The study population was described by the following characteristics: sex, gestational age, and comorbidity status. Comorbidity status was determined by the presence of a comorbidity *ICD-10-CA* discharge diagnosis code on a hospital encounter from birth to 2 years. Additionally, procedural codes were used for children with a complex chronic condition (eg, gastrostomy), following the Canadian Institute for Health Information methods.^24^ Comorbidities were not mutually exclusive and included complex chronic condition,^24,25^ chronic lung disease or bronchopulmonary dysplasia arising from the perinatal period,^18^ congenital heart disease,^26^ neurologic impairment,^27^ immunodeficiency, and trisomy 21.^26^

### Equity Stratifiers

Health inequalities were determined for the following equity stratifiers: sex (male or female), geography (rural or urban residence), and material resources quintile. Race and ethnicity data are not available through Ontario health administrative databases. The primary residence location was assessed using the Rurality Index of Ontario, which defines neighborhoods into urban (score of <40) or rural (score of ≥40).^28^ The Rurality Index of Ontario score is calculated using community population size, density, and travel time to the nearest basic and advanced health care referral centers. We used the 2008 version of the rurality index, which is the most recent version available and currently used by the Ontario government. Material resources is 1 of 4 dimensions of the Ontario Marginalization Index that is used as a comprehensive area-based measure of socioeconomic status and poverty at the neighborhood level.^29^ It is based on level of education (proportion of population 25 years or older without a certificate, diploma, or degree), family structure (proportion of lone-parent families), housing quality (proportion of homes needing a major repair), employment (proportion of population 15 years or older who are unemployed), and income (proportion of population below the low-income cutoff and proportion receiving government transfer payments). Material resources quintiles were created at the dissemination area level and assigned to individual records, with quintile 1 representing the most resourced (ie, least deprived) and quintile 5 representing the least resourced (ie, most deprived). The material resources index is available for 2001, 2006, 2011, 2016, and 2021 for tracking changes over time.

### Outcomes

Bronchiolitis outcomes included the population-based bronchiolitis ED visit and hospitalization rate. Outcomes were estimated annually (April 1 to March 31) and for the entire study period (April 1, 2004, to March 31, 2022). The population-based ED visit rate was estimated per 1000 person-years with 95% CIs. The numerator was the total number of bronchiolitis ED visits for all children younger than 2 years, and the denominator was the number of person-years contributed by all children younger than 2 years in that period. The hospitalization rate was estimated per 1000 person-years with 95% CIs. The numerator was the total number of bronchiolitis hospitalizations for all children younger than 2 years, and the denominator was the number of person-years contributed by all children younger than 2 years in that period. Crude rates were estimated because the focus was on understanding the health system and population burden of bronchiolitis hospital use.

### Statistical Analysis

We used joinpoint regression analysis to examine temporal trends in bronchiolitis ED visit rates and hospitalization rates by sex, residence location, and material resources quintile from April 1, 2004, to March 31, 2020.^30^ The joinpoint regression software calculates the number and temporal location of points representing a statistically significant change in trend (ie, a joinpoint).^31^ We used Joinpoint, version 5.0.2 (National Cancer Institute) to run the joinpoint regression models, starting with a model with 0 joinpoints (a linear relationship) and testing whether 1 or more joinpoints should be added to the final model. We selected the grid search method for fitting the model and the permutation test (n = 4499 permutations) for determining the optimal number of joinpoints. We selected log transformation to calculate annual average percent change (AAPC), the average rate of change in ED rate (or hospitalization rate) per year during the study period. The AAPC was tested against the null hypothesis that the percent change was zero (no increase or decrease over time). A pairwise comparison between trends (with the null hypothesis that the 2 lines are parallel) was performed to examine whether the rate of change (AAPC) differed between groups. A 95% CI around the absolute difference in the AAPC between groups (eg, male vs female) that includes 0 indicates no increase or decrease in the inequality gap over time between groups. Statistical significance was defined as *P* < .05; all statistical tests were 2-sided.

## Results

The study cohort included 2 921 573 children, of whom 1 499 485 (51.3%) were male and 1 422 088 (48.7%) were female, 545 378 (18.7%) were preterm and/or had a comorbidity, 105 189 (3.6%) had a chronic complex condition, and 2 619 139 (89.6%) lived in an urban location (Table 1). Bronchiolitis ED visits occurred at 214 hospitals (185 [86.4%] community and 29 [13.6%] pediatric or teaching) and hospitalizations at 151 hospitals (134 [88.7%] community and 17 (11.2%) pediatric or teaching).

**Table 1.** Characteristics of the Cohort

Characteristic	All children (birth to 2 y), No. (%) (n = 2 921 573)
Sex	
Female	1 422 088 (48.7)
Male	1 499 485 (51.3)
Gestational age, wk	
22-27	10 768 (0.4)
28-32	28 454 (1.0)
33-36	171 641 (5.9)
≥37	2 444 683 (83.7)
Missing	266 027 (9.1)
Chronic complex	105 189 (3.6)
Chronic lung disease	6817 (0.2)
Congenital heart disease	35 206 (1.2)
Neurologic impairment	13 972 (0.5)
Comorbidity and/or preterm	545 378 (18.7)
Residence	
Urban	2 619 139 (89.6)
Rural	289 542 (9.9)
Missing	12 892 (0.4)
Material resources quintile	
1 (Least deprived)	576 490 (19.7)
2	550 419 (18.8)
3	540 734 (18.5)
4	541 917 (18.5)
5 (Most deprived)	662 344 (22.7)
Missing	49 669 (1.7)

### ED Visit Rates

**Figure 1.**
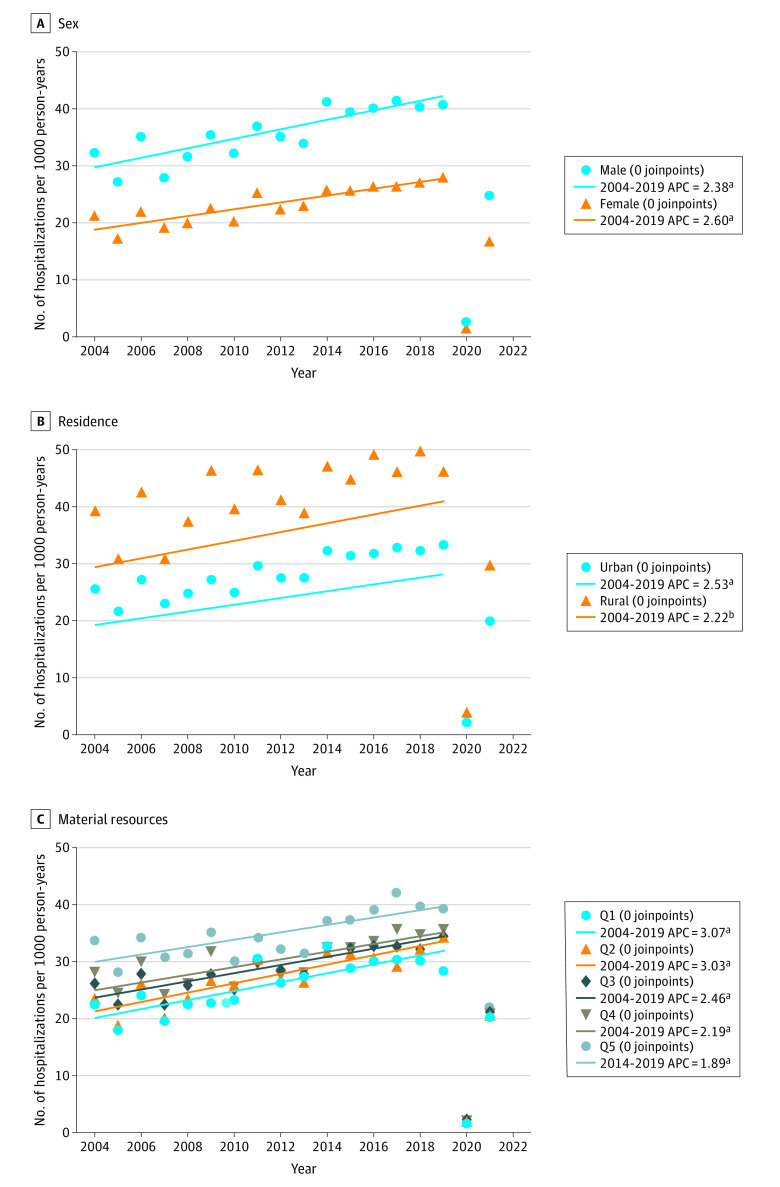
Trends in Bronchiolitis Emergency Department Visit Rates Among Equity Stratifiers, 2004-2005 to 2021-2022 Emergency department visit rates are per 1000 person-years in children younger than 2 years are shown. Symbols are the crude annual (April 1 to March 31) rates from April 1, 2004, to March 31, 2022, among groups. Trends were quantified using the Joinpoint regression program, version 5.0.5 (National Cancer Institute). Annual percent change (APC) was estimated for years 2004-2005 to 2019-2020. There was no evidence of a significant joinpoint for sex, residence location, or material resources quintile (Q), indicating that the observed increases in emergency department visit rates were linear. ^a^The average APC was significantly different from zero at *P* < .001. ^b^The average APC was significantly different from zero at *P* < .01.

During the study period, there were 141 045 bronchiolitis ED visits for an overall population-based ED visit rate of 27.8 (95% CI, 27.6-27.9) per 1000 person-years. eTables 1-3 in Supplement 1 provide the annual ED visit rates by equity stratifier and rate ratios with 95% CIs. The ED visit rates were lower for girls than boys, greater for children living in a rural location than an urban location, and greater for children with the least material resources. The ED visit rates increased from years 2004-2005 to 2019-2020. The 2020-2021 rates saw a marked reduction associated with the pandemic; in 2021-2022 rates showed an increase but not to prepandemic rates. There was no evidence of a significant joinpoint for sex, residence location, or material deprivation quintile, indicating that the observed increases in ED visit rates were linear over time to 2019-2020 (Figure 1).

The ED visit rates increased over time for both boys (AAPC, 2.38 [95% CI, 1.48-3.30] visits per 1000 person-years; *P* < .001) and girls (AAPC, 2.60 [95% CI, 1.76-3.45]) visits per 1000 person-years; *P* < .001), with no significant between-group difference in the rate of change (0.22 [95% CI, −0.92 to 1.35] visits per 1000 person years; *P* = .71) (Figure 1 and Table 2).

**Table 2.** Trends in Bronchiolitis ED Visit Rates by Equity Stratifiers, 2004-2005 to 2019-2020^a^

Characteristic	No. (%) of ED visits (n = 141 045)^b^	ED visit rate, % (95% CI)		AAPC (95% CI)^c^	Between-group difference	
		2004-2005	2019-2020		AAPC, % (95% CI)	*P* value
Sex						
Male	87 008 (61.7)	32.4 (31.5 to 33.4)	40.8 (39.8 to 41.9)	2.38 (1.48 to 3.30)^d^	1.0 [Reference]	NA
Female	54 037 (38.3)	21.4 (20.6 to 22.2)	28.1 (27.3 to 29.0)	2.60 (1.76 to 3.45)^d^	0.22 (−0.92 to 1.35)	.71
Residence						
Urban	120 685 (85.6)	25.6 (25.0 to 26.3)	33.4 (32.7 to 34.1)	2.53 (1.72 to 3.34)^d^	1.0 [Reference]	NA
Rural	20 102 (14.3)	39.2 (37.0 to 41.5)	46.1 (43.6 to 48.7)	2.22 (0.94 to 3.52)^e^	−0.31 (−1.70 to 1.09)	.67
Missing	258 (0.2)	NA	NA	NA	NA	NA
Material resources quintile						
1 (Least deprived)	24 392 (17.3)	22.5 (21.3 to 23.7)	28.4 (27.1 to 29.8)	3.07 (1.83 to 4.32)^d^	1.0 [Reference]	NA
2	24 461 (17.3)	23.6 (22.3 to 25.0)	34.3 (32.8 to 35.9)	3.03 (1.92 to 4.15)^d^	−0.04 (−1.57 to 1.49)	.96
3	25 364 (18.0)	26.2 (24.8 to 27.7)	34.5 (32.9 to 36.1)	2.46 (1.63 to 3.31)^d^	−0.60 (−1.97 to 0.77)	.39
4	26 519 (18.8)	28.2 (26.8 to 29.8)	35.7 (34.1 to 37.4)	2.19 (1.21 to 3.18)^d^	−0.88 (−2.33 to 0.57)	.23
5 (Most deprived)	37 490 (26.6)	33.7 (32.2 to 35.2)	39.4 (37.9 to 41.1)	1.89 (1.00 to 2.79)^d^	−1.17 (−2.57 to 0.22)	.10
Missing	2819 (2.0)	NA	NA	NA	NA	NA

Abbreviations: AAPC, average annual percentage change; ED, emergency department; NA, not applicable.

^a^ The annual percentage change over the period 2004-2020 is identical to the average for all subgroups.

^b^ The percentages in each row were calculated using the total number as the denominator.

^c^ Trends were quantified using the Joinpoint regression program, version 5.0.2 (National Cancer Institute).

^d^ The AAPC was significantly different from zero at *P* < .001.

^e^ The AAPC was significantly different from zero at *P* < .01.

The ED visit rates increased over time for both children with an urban (AAPC, 2.53 [95% CI 1.72-3.34] visits per 1000 person-years; *P* < .001) and rural (AAPC, 2.22 [95% CI, 0.94-3.52]) visits per 1000 person-years; *P* = .002) residence, with no significant between-group difference in the rate of change (−0.31 [95% CI, −1.70 to 1.09] visits per 1000 person-years; *P* = .67) (Figure 1 and Table 2).

The ED visit rates increased for all material resources quintiles, with the greatest increase for the group with greatest material resources (quintile 1) (AAPC, 3.07 [95% CI, 1.83-4.32] visits per 1000 person- years; *P* < .001) and lowest for the group with lowest material resources (quintile 5) (1.89 [95% CI, 1.00-2.79] visits per 1000 person-years; *P* < .001) (Figure 1 and Table 2). However, there were no statistically significant between-group differences in the rate of change for each quintile compared with the group with the greatest material resources (eg, quintile 5 vs 1: −1.17; 95% CI, −2.57 to 0.22; *P* = .10) (Table 2).

### Hospitalization

**Figure 2.**
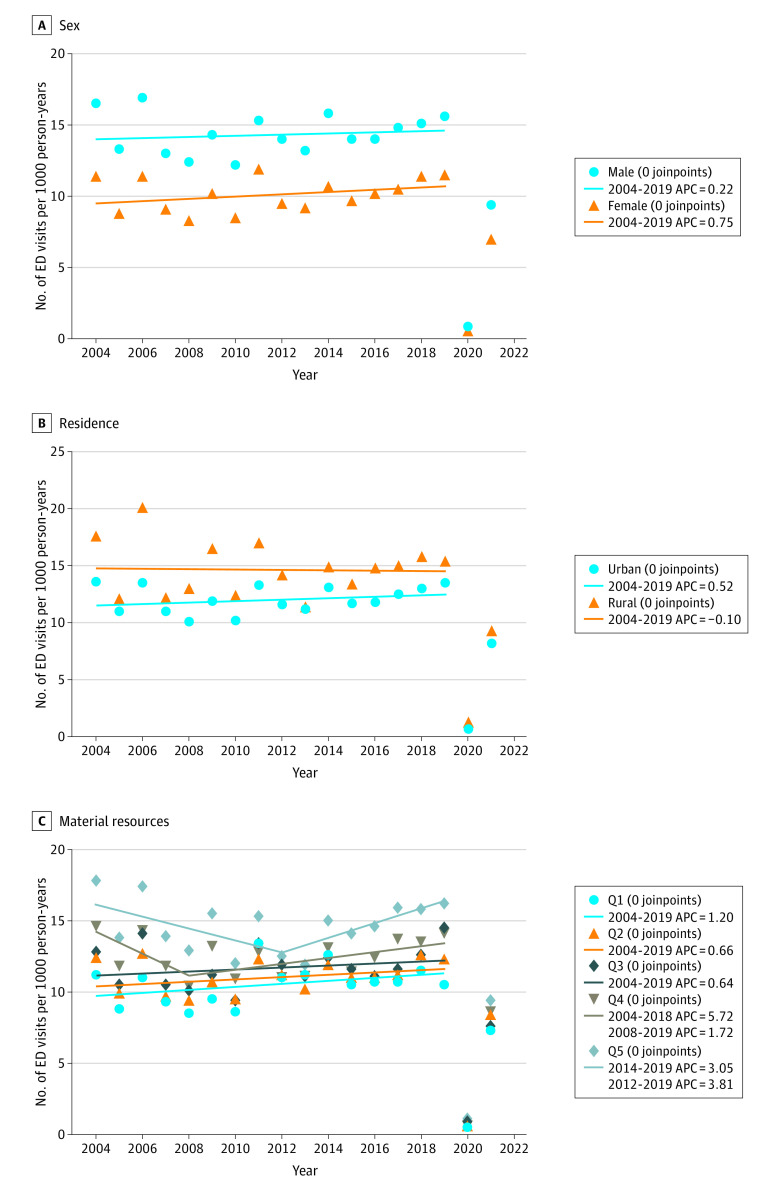
Trends in Bronchiolitis Hospitalization Visit Rates Among Equity Stratifiers, 2004-2005 to 2021-2022 Hospitalization rates are per 1000 person-years in children younger than 2 years are shown. Symbols are the crude annual (April 1 to March 31) rates from April 1, 2004, to March 31, 2022, among groups. Trends were quantified using the Joinpoint regression program, version 5.0.5 (National Cancer Institute). Annual percent change (APC) was estimated for years 2004-2005 to 2019-2020. There was no evidence of a significant joinpoint for sex and residence location. For material resources, in quintile (Q) 4, there was an initial decrease in hospitalization rate from 2004 to 2008 (trend 1: APC, −5.72; 95% CI, −14.10 to 3.49; *P* = .19) followed by an increase from 2008 to 2019 (trend 2: APC, 1.72; 95% CI, −0.28 to 3.76; *P* = .09). In Q5, there was an initial decrease in hospitalization rate from 2004 to 2012 (trend 1: APC, −3.05; 95% CI, −6.51 to 0.53; *P* = .09) followed by an increase from 2012 to 2019 (trend 2: APC, 3.81; 95% CI, −0.70 to 8.53; *P* = .09). However, these trends for Q4 and Q5 were not statistically significant. The average annual percent change (AAPC) for each of the Q1 to Q5 subgroups was not significantly different from zero at the α = .05 level. The AAPC difference between Q2 to Q5 and referent Q1 were not significantly different from zero at the α = .05 level.

There were 58 215 bronchiolitis hospitalizations during the study period for an overall hospitalization rate of 11.4 (95% CI, 11.4-11.6) hospitalizations per 1000 person-years. As displayed in Figure 2, there was no evidence of a significant joinpoint for sex and residence location, indicating that the observed stable hospitalization rates were linear over time to 2019-2020. There was 1 joinpoint for material resources quintiles 4 and 5, which was not statistically significant. Similar to ED rates, hospitalization rates in 2020-2021 saw a marked reduction associated with the pandemic, and then the 2021-2022 rates showed an increase, but not to prepandemic rates.

Hospitalization rates were lower for girls compared with boys (eTables 4-6 in Supplement 1). Hospitalization rates were stable over time for both boys (AAPC, 0.22 [95% CI, −0.94 to 1.40] visits per 1000 person-years) and girls (AAPC, 0.75 [95% CI, −0.60 to 2.12] visits per 1000 person-years), with no significant between-group difference in the rate of change (0.53 [95% CI, −1.11 to 2.17] visits per 1000 person-years; *P* = .53) (Figure 2 and Table 3).

**Table 3.** Trends in Bronchiolitis Hospitalization Rates by Equity Stratifiers, 2004-2005 to 2019-2020

Characteristic	No. (%) of hospitalizations (n = 58 215)^a^	Hospitalization rate (95% CI)		AAPC (95% CI)^b^	Between-group difference	
		2004-2005	2019-2020		AAPC, % (95% CI)	*P* value
Sex						
Male	34 885 (59.9)	16.5 (15.9 to 17.2)	15.6 (15.0 to 16.3)	0.22 (−0.94 to 1.40)	1.0 [Reference]	NA
Female	23 330 (40.1)	11.4 (10.8 to 11.9)	11.5 (11.0 to 12.1)	0.75 (−0.60 to 2.12)	0.53 (−1.11 to 2.17)	.53
Residence						
Urban	51 129 (87.8)	13.6 (13.2 to 14.1)	13.5 (13.0 to 13.9)	0.52 (−0.65 to 1.70)	1.0 [Reference]	NA
Rural	6987 (12.0)	17.6 (16.1 to 19.2)	15.4 (14.0 to 17.0)	−0.10 (−1.95 to 1.79)	−0.62 (−2.63 to 1.40)	.55
Missing	99 (0.2)	NA	NA	NA	NA	NA
Material resources quintile						
1 (Least deprived)	9823 (16.9)	11.2 (10.4 to 12.1)	10.5 (9.7 to 11.4)	1.02 (−0.44 to 2.51)	1.0 [Reference]	NA
2	9880 (17.0)	12.4 (11.4 to 13.4)	12.3 (11.4 to 13.3)	0.66 (−0.53 to 1.87)	−0.36 (−2.10 to 1.38)	.69
3	10 291 (17.7)	12.8 (11.8 to 13.8)	14.5 (13.5 to 15.5)	0.64 (−0.76 to 2.06)	−0.38 (−2.25 to 1.48)	.69
4	10 994 (18.9)	14.6 (13.5 to 15.7)	14.1 (13.1 to 15.2)	−0.32 (−2.84 to 2.27)	−1.34 (−4.23 to 1.55)	.36
5 (Most deprived)	15 757 (27.1)	17.8 (16.8 to 18.9)	16.2 (15.2 to 17.2)	0.09 (−2.41 to 2.65)	−0.93 (−3.80 to 1.93)	.52
Missing	1470 (2.5)	NA	NA	NA	NA	NA

Abbreviations: AAPC, average annual percentage change; NA, not applicable.

^a^ The percentages in each row were calculated using the total number as the denominator.

^b^ Trends were quantified using the Joinpoint regression program, version 5.0.2 (National Cancer Institute).

Hospitalization rates were greater for children living in a rural location compared with an urban location (eTable 5 in Supplement 1). Hospitalization rates were stable over time for both children with an urban (AAPC, 0.52 [95% CI, −0.65 to 1.70] visits per 1000 person-years) and rural (AAPC, −0.10 [95% CI, −1.95 to 1.79] visits per 1000 person-years) residence, with no significant between-group difference in the rate of change (−0.62 [95% CI, −2.63 to 1.40] visits per 1000 person-years; *P* = .55) (Figure 2 and Table 2).

During the study period, the hospitalization rate was lowest for the group with the greatest material resources (quintile 1) (eTable 6 in Supplement 1). The hospitalization rate was stable for all material resources quintiles, and there were no statistically significant between-group differences in the rate of change for each quintile compared with the group with greatest material resources (eg, quintile 5 vs 1: −0.93; 95% CI −3.80 to 1.93; *P* = .52) (Figure 2 and Table 2).

## Discussion

This population-based cohort study examined trends in bronchiolitis-related ED visit and hospitalization rates by sociodemographic factors in a universally funded health care system from 2004 to 2022. Crude rates were highest for boys, rural residents, and those with the least material resources. We found no significant differences in the AAPC in the ED visit and hospitalization rates between groups based on sex, residence, and material resources. These data indicate that health inequalities among children with bronchiolitis in Ontario (based on an examination of sex, location of residence, and material resources as potential equity stratifiers) did not improve over time. Although health inequalities also did not get worse over time, the existence of inequalities suggests that we need to reallocate effort and resources to those groups experiencing poorer outcomes to improve children’s health.

Few previous studies^15,20^ have examined trends in bronchiolitis inequalities to examine whether the inequality gap is narrowing or widening. Fujiogi et al^15^ examined bronchiolitis hospitalizations in the US from 2000 to 2016 in a serial cross-sectional analysis using the Kids’ Inpatient Database. In contrast to our data source, the Kids’ Inpatient Database does not contain data on the denominator of at-risk children. Thus, Fujiogi et al^15^ could not determine population-based hospitalization rates by equity stratifiers to examine temporal trends. Chung et al^20^ reported increasing hospitalization rates in Scotland from 2001 to 2016, with a hospitalization rate of 45 per 1000 children in the most deprived group vs 23 per 1000 in the least deprived group. In contrast to our findings, Chung et al^20^ found a greater increase in the hospitalization rate in the most deprived compared with the least deprived group during 5 years, comparing 2011 with 2015. Other studies^16,19,20,32,33,34,35^ quantify inequalities at one time point rather than examining trends in inequalities. These studies found higher rates of bronchiolitis ED visits or hospitalization based on race, Indigenous status, material deprivation, health care insurance provider, and maternal age in the US, Canada, New Zealand, and the UK.

Health system or bronchiolitis-specific interventions implemented during the study period might have affected bronchiolitis ED or admission rates. Between 2001 and 2006, the Ontario government introduced new primary care models characterized by group-based care, physician remuneration by salary (vs fee for service), pay for performance, and a requirement for after-hours services.^36^ These primary care interventions are aimed at increasing primary care access and reducing ED visits and hospitalizations. However, we observed an increase in bronchiolitis-related ED visits over time.

For this 18-year study, we observed persistent health inequalities in bronchiolitis ED visit and hospitalization rates. Several underlying factors may contribute to the sustained inequality. Disparities in bronchiolitis outcomes based on sex have been reported in previous studies.^11,13,15,37^ Both biological (ie, smaller airways in males and hormonal and immunologic difference) and possibly social factors have been suggested as explanations for higher rates of ED visits, hospitalizations, and severity of illness in males.^37,38,39^ Residents of rural areas often face chronic health care infrastructure limitations from primary to tertiary care, leading to inadequate health care access and/or delayed medical care.^40^ Socioeconomic disparities, including material deprivation, remain a pervasive issue, creating barriers to access and timely use of health care services.^41,42^ Emerging preventive interventions hold promise in addressing inequalities in sex, residence location, material resources, and other equity stratifiers (eg, race and/or ethnicity). Vaccination for RSV and new RSV monoclonal antibodies (eg, nirsevimab) could prevent a substantial proportion of RSV-related bronchiolitis and, if implemented equitably, may narrow the health inequalities gap.^43,44,45,46^ Evaluation of inequalities after implementation is important because it is possible that these interventions could also increase disparities in bronchiolitis outcomes if access to them is not equitable.

### Limitations

Several limitations to this study are important to consider. Data on individuals’ race, ethnicity, or indigeneity were unavailable in the data source; thus, we could not describe trends in health inequalities based on these important strata. Children with comorbidities are at risk for severe bronchiolitis and may not have been equally distributed across the groups examined. Rurality was measured using the most recent version of the rurality index of Ontario from 2008. It is possible that some jurisdictions moved from rural to urban since 2008. Such misclassification could bias the results toward finding less inequality. Our study focused on ED visits and hospitalization outcomes. Further studies examining inequalities in other important outcomes, such as length of stay and intensive care unit admission, are needed. Lastly, the findings of this study may not be generalizable to jurisdictions without universal health care coverage.

## Conclusions

This population-based cohort study in a universally funded health care system found that inequalities in bronchiolitis ED and hospitalization rates from 2004 to 2022 did not improve. Future research should evaluate whether emerging preventive interventions, such as RSV vaccination and newer monoclonal antibodies, narrow the bronchiolitis inequalities gap.

## Notes

Supplement 1. **eMethods.** Supplemental Methods **eTable 1.** Trends in Bronchiolitis Emergency Department Visit Rates Among Equity Stratifiers: Female vs. Male, 2004-2005 to 2021-2022 **eTable 2.** Trends in Bronchiolitis Emergency Department Visit Rates Among Equity Stratifiers: Rural vs. Urban, 2004-2005 to 2021-2022 **eTable 3.** Trends in Bronchiolitis Emergency Department Visit Rates Among Equity Stratifiers: Material Resources Quintile, 2004-2005 to 2021-2022 **eTable 4.** Trends in Bronchiolitis Hospitalization Rates Among Equity Stratifiers: Female vs. Male, 2004-2005 to 2021-2022 **eTable 5.** Trends in Bronchiolitis Hospitalization Rates Among Equity Stratifiers: Rural vs. Urban, 2004-2005 to 2021-2022 **eTable 6.** Trends in Bronchiolitis Hospitalization Rates Among Equity Stratifiers: Material Resources Quintile, 2004-2005 to 2021-2022 **eAppendix.** Canadian Paediatric Inpatient Research Network Members

Supplement 2.
Data Sharing Statement
