# Supplementary material for: Sociodemographic Factors and Trends in Bronchiolitis-Related Emergency Department Visit and Hospitalization Rates
Source: JAMA Netw Open. 2024 Apr 29;7(4):e248976. doi: 10.1001/jamanetworkopen.2024.8976 (PMC11059049; doi:10.1001/jamanetworkopen.2024.8976)
Supplement: Supplement 1. — eMethods. Supplemental Methods eTable 1. Trends in Bronchiolitis Emergency Department Visit Rates Among Equity Stratifiers: Female vs. Male, 2004-2005 to 2021-2022 eTable 2. Trends in Bronchiolitis Emergency Department Visit Rates Among Equity Stratifiers: Rural vs. Urban, 2004-2005 to 2021-2022 eTable 3. Trends in Bronchiolitis Emergency Department Visit Rates Among Equity Stratifiers: Material Resources Quintile, 2004-2005 to 2021-2022 eTable 4. Trends in Bronchiolitis Hospitalization Rates Among Equity Stratifiers: Female vs. Male, 2004-2005 to 2021-2022 eTable 5. Trends in Bronchiolitis Hospitalization Rates Among Equity Stratifiers: Rural vs. Urban, 2004-2005 to 2021-2022 eTable 6. Trends in Bronchiolitis Hospitalization Rates Among Equity Stratifiers: Material Resources Quintile, 2004-2005 to 2021-2022 eAppendix. Canadian Paediatric Inpatient Research Network Members [file jamanetwopen-e248976-s001.pdf]

Supplementary Online Content

Mahant S, Borkhoff CM, Parkin PC, et al. Sociodemographic factors and trends in bronchiolitis-related emergency department visit and hospitalization rates. *JAMA Netw Open*. 2024;7(5):e248976. doi: 10.1001/jamanetworkopen.2024

eMethods. Supplemental Methods
eTable 1. Trends in Bronchiolitis Emergency Department Visit Rates Among Equity Stratifiers: Female vs. Male, 2004-2005 to 2021-2022
eTable 2. Trends in Bronchiolitis Emergency Department Visit Rates Among Equity Stratifiers: Rural vs. Urban, 2004-2005 to 2021-2022
eTable 3. Trends in Bronchiolitis Emergency Department Visit Rates Among Equity Stratifiers: Material resources quintile, 2004-2005 to 2021-2022
eTable 4. Trends in Bronchiolitis Hospitalization Rates Among Equity Stratifiers: Female vs. Male, 2004-2005 to 2021-2022
eTable 5. Trends in Bronchiolitis Hospitalization Rates Among Equity Stratifiers: Rural vs. Urban, 2004-2005 to 2021-2022
eTable 6. Trends in Bronchiolitis Hospitalization Rates Among Equity Stratifiers: Material resources quintile, 2004-2005 to 2021-2022
eAppendix. Canadian Paediatric Inpatient Research Network Members

This supplementary material has been provided by the authors to give readers additional information about their work.

© 2024 Mahant S et al. *JAMA Network Open.*

eMethods. Supplemental Methods

Databases

Administrative data housed and analyzed at ICES were used in this study. A unique identifier, the ICES Key Number (IKN), is common to all datasets at ICES and used to link individuals.

Registered Persons Database (RPDB)

The Registered Persons Database (RPDB) is maintained by the Ministry of Health and Long-Term Care (MOHLTC) in Ontario, Canada. It is a population-based registry that is used to manage the Ontario Health Insurance Plan, which is the publicly funded health care services plan. RPDB contains a listing of the unique health numbers that have been issued to individuals eligible for health coverage. It contains demographic information included an individual's date of birth, sex, address (postal codes), data of death, and contains data on changes in health insurance coverage eligibility.

National Ambulatory Care Reporting System (NACRS)

NACRS captures data on patient visits to ambulatory care facilities, including hospital and community facilities. Encounters that are captured are day surgery, outpatient clinic visits, and emergency department visits. Data elements include patient demographic information, clinical data on the patients' diagnoses, the procedures they undergo, and the physicians who care for them. NACRS is coded using the International Classification of Diseases (ICD), Version 10 since 2002. Procedures are coded using the Canadian Classification of Interventions (CCI).

Discharge Abstract Data (DAD)

The DAD is a national database that contains patient-level administrative, clinical and demographic information on all hospital discharges, whether they be inpatient acute, chronic or rehabilitation. Information is abstracted from hospital records. Each record in the database corresponds to a single hospitalization. Data elements include patient demographic information, clinical data on the patients' diagnoses, the procedures they undergo, and the physicians who care for them. There is also information on the institutions that they are treated at, length of hospital stay, and their disposition at discharge. DAD is coded using the International Classification of Diseases (ICD), Version 10 since 2002. Procedures are coded using the Canadian Classification of Interventions (CCI), since 2002.

MOMBABY
The MOMBABY database contains 100% of Ontario inpatient birth admissions records from the Discharge Abstract Database (DAD) for mothers and their newborns delivered from 2002. It links mothers and their newborns deterministically based on the maternal and newborn chart number. Each record contains the unique encrypted health care number, age, sex of the individual, the data of admission and up to 25 diagnosis codes identified with the ICD coding system.

© 2024 Mahant S et al. *JAMA Network Open.*

eTable 1. Trends in Bronchiolitis Emergency Department Visit Rates Among Equity Stratifiers: Female vs. Male, 2004-2005 to 2021-2022

	Total Person-Years, No. 5,082,191						
	Total ED Visits, No. 141,045						
	Male			Female			
Year	ED Visits, No.	Person-Years, No.	ED Visit Rate, No. per 1000 person-years (95% CI)	ED Visits, No.	Person-Years, No.	ED Visit Rate, No. per 1000 person-years (95% CI)	Rate Ratio = Female/Male (95% CI)
2004	4,584	141,382	32.4 (31.5, 33.4)	2,860	133,860	21.4 (20.6, 22.2)	0.66 (0.63, 0.69)
2005	3,908	143,197	27.3 (26.4, 28.2)	2,351	135,216	17.4 (16.7, 18.1)	0.64 (0.60, 0.67)
2006	5,060	143,608	35.2 (34.3, 36.2)	2,998	135,939	22.1 (21.3, 22.9)	0.63 (0.60, 0.66)
2007	4,079	145,811	28.0 (27.1, 28.9)	2,673	138,464	19.3 (18.6, 20.1)	0.69 (0.66, 0.73)
2008	4,689	147,949	31.7 (30.8, 32.6)	2,836	140,800	20.1 (19.4, 20.9)	0.64 (0.61, 0.67)
2009	5,228	147,092	35.5 (34.6, 36.5)	3,171	139,652	22.7 (21.9, 23.5)	0.64 (0.61, 0.67)
2010	4,613	142,871	32.3 (31.4, 33.2)	2,757	135,467	20.4 (19.6, 21.1)	0.63 (0.60, 0.66)
2011	5,326	143,827	37.0 (36.0, 38.0)	3,466	136,626	25.4 (24.5, 26.2)	0.69 (0.66, 0.72)
2012	5,160	146,762	35.2 (34.2, 36.1)	3,133	139,300	22.5 (21.7, 23.3)	0.64 (0.61, 0.67)
2013	4,968	146,235	34.0 (33.0, 34.9)	3,206	138,822	23.1 (22.3, 23.9)	0.68 (0.65, 0.71)
2014	5,982	144,928	41.3 (40.2, 42.3)	3,575	137,976	25.9 (25.1, 26.8)	0.63 (0.60, 0.65)
2015	5,735	145,037	39.5 (38.5, 40.6)	3,557	138,030	25.8 (24.9, 26.6)	0.65 (0.62, 0.68)
2016	5,849	145,436	40.2 (39.2, 41.3)	3,653	137,960	26.5 (25.6, 27.4)	0.66 (0.63, 0.69)
2017	6,045	145,514	41.5 (40.5, 42.6)	3,656	137,847	26.5 (25.7, 27.4)	0.64 (0.61, 0.67)
2018	5,875	145,471	40.4 (39.4, 41.4)	3,738	137,600	27.2 (26.3, 28.1)	0.67 (0.65, 0.70)
2019	5,933	145,436	40.8 (39.8, 41.9)	3,887	138,140	28.1 (27.3, 29.0)	0.69 (0.66, 0.72)
2020	404	143,853	2.8 (2.5, 3.1)	227	136,885	1.7 (1.5, 1.9)	0.59 (0.50, 0.70)
2021	3,570	143,115	24.9 (24.1, 25.8)	2,293	136,084	16.9 (16.2, 17.6)	0.68 (0.64, 0.71)
All years	87,008	2,607,523	33.4 (33.2, 33.6)	54,037	2,474,668	21.8 (21.7, 22.0)	
AAPC, % (95% CI)[b]			2.38 (1.48, 3.30)[c]			2.60 (1.76, 3.45)[c]	
% difference AAPC (95% CI)			ref			0.22 (-0.92, 1.35)	

[a]Data are expressed as No. (%). The percentage in each row were calculated using the total number as the denominator.

[b]Trends were quantified using version 5.0.2 of the Joinpoint Regression program (National Cancer Institute). No joinpoints were identified. AAPC was estimated for years 2004-2005 to 2019-2020.

The annual percentage change over the period 2004-2019 is identical to the average for both subgroups.

[c]The AAPC was significantly different from zero at p<0.001.

AAPC, Average Annual Percentage Change

© 2024 Mahant S et al. *JAMA Network Open.*

eTable 2. Trends in Bronchiolitis Emergency Department Visit Rates Among Equity Stratifiers: Rural vs. Urban, 2004-2005 to 2021-2022

	**Total Person-Years, No.** 5,082,191						
	**Total ED Visits, No.** 141,045						
	**Urban**			**Rural**			
**Year**	**ED Visits, No.**	**Person-Years, No.**	**ED Visit Rate, No. per 1000 person-years (95% CI)**	**ED Visits, No.**	**Person-Years, No.**	**ED Visit Rate, No. per 1000 person-years (95% CI)**	**Rate Ratio = Rural/Urban (95% CI)**
2004	6,269	244,519	25.6 (25.0, 26.3)	1,162	29,650	39.2 (37.0, 41.5)	1.53 (1.43, 1.63)
2005	5,367	249,101	21.6 (21.0, 22.1)	885	28,745	30.8 (28.8, 32.9)	1.43 (1.33, 1.54)
2006	6,847	250,862	27.3 (26.7, 28.0)	1,205	28,376	42.5 (40.1, 44.9)	1.56 (1.46, 1.66)
2007	5,859	255,248	23.0 (22.4, 23.6)	886	28,793	30.8 (28.8, 32.9)	1.34 (1.25, 1.44)
2008	6,428	259,200	24.8 (24.2, 25.4)	1,091	29,290	37.3 (35.1, 39.5)	1.50 (1.41, 1.60)
2009	7,036	257,312	27.3 (26.7, 28.0)	1,353	29,170	46.4 (43.9, 48.9)	1.70 (1.60, 1.80)
2010	6,241	249,751	25.0 (24.4, 25.6)	1,120	28,319	39.6 (37.3, 41.9)	1.58 (1.48, 1.69)
2011	7,456	251,633	29.7 (29.0, 30.3)	1,327	28,514	46.5 (44.1, 49.1)	1.57 (1.48, 1.67)
2012	7,100	256,872	27.6 (27.0, 28.3)	1,189	28,882	41.2 (38.9, 43.6)	1.49 (1.40, 1.59)
2013	7,041	255,915	27.5 (26.9, 28.2)	1,121	28,831	38.9 (36.6, 41.2)	1.41 (1.33, 1.51)
2014	8,198	254,057	32.3 (31.6, 33.0)	1,337	28,375	47.1 (44.6, 49.7)	1.46 (1.38, 1.55)
2015	8,035	254,733	31.5 (30.9, 32.2)	1,240	27,664	44.8 (42.4, 47.4)	1.42 (1.34, 1.51)
2016	8,138	255,348	31.9 (31.1, 32.6)	1,339	27,279	49.1 (46.5, 51.8)	1.54 (1.45, 1.63)
2017	8,401	255,027	32.9 (32.2, 33.7)	1,265	27,459	46.1 (43.6, 48.7)	1.42 (1.34, 1.51)
2018	8,205	254,236	32.3 (31.6, 33.0)	1,382	27,826	49.7 (47.1, 52.4)	1.54 (1.45, 1.63)
2019	8,512	254,791	33.4 (32.7, 34.1)	1,279	27,748	46.1 (43.6, 48.7)	1.38 (1.30, 1.47)
2020	522	252,696	2.1 (1.9, 2.3)	103	27,158	3.8 (3.1, 4.6)	1.84 (1.48, 2.28)
2021	5,030	250,998	20.0 (19.5, 20.6)	818	27,434	29.8 (27.8, 31.9)	1.49 (1.38, 1.60)
All years	120,685	4,562,301	26.5 (26.3, 26.6)	20,102	509,513	39.5 (38.9, 40.0)	
AAPC, % (95% CI)[b]			2.53 (1.72, 3.34)[c]			2.22 (0.94, 3.52)[d]	
% difference AAPC (95% CI)			ref			-0.31 (-1.70, 1.09)	

[a]Data are expressed as No. (%). The percentage in each row were calculated using the total number as the denominator.
[b]Trends were quantified using version 5.0.2 of the Joinpoint Regression program (National Cancer Institute). No joinpoints were identified.
AAPC was estimated for years 2004-2005 to 2019-2020.
The annual percentage change over the period 2004-2019 is identical to the average for both subgroups.
[c]The AAPC was significantly different from zero at p<0.001.
[d]The AAPC was significantly different from zero at p=0.002.
AAPC, Average Annual Percentage Change

© 2024 Mahant S et al. *JAMA Network Open.*

eTable 3. Trends in Bronchiolitis Emergency Department Visit Rates Among Equity Stratifiers: Material resources quintile, 2004-2005 to 2021-2022

	**Total Person-Years, No.** 5,082,191					
	**Total ED Visits, No.** 141,045					
	**ED Visits, No.** 24,392	**ED Visits, No.** 24,461	**ED Visits, No.** 25,364	**ED Visits, No.** 26,519	**ED Visits, No.** 37,490	
	**Q1**	**Q2**	**Q3**	**Q4**	**Q5**	
**Year**	**ED Visit Rate, No. per 1000 person-years (95% CI)**	**ED Visit Rate, No. per 1000 person-years (95% CI)**	**ED Visit Rate, No. per 1000 person-years (95% CI)**	**ED Visit Rate, No. per 1000 person-years (95% CI)**	**ED Visit Rate, No. per 1000 person-years (95% CI)**	**Rate Ratio = Q5/Q1 (95% CI)**
2004	22.5 (21.3, 23.7)	23.6 (22.3, 25.0)	26.2 (24.8, 27.7)	28.2 (26.8, 29.8)	33.7 (32.2, 35.2)	1.50 (1.40, 1.61)
2005	18.0 (16.9, 19.1)	18.9 (17.7, 20.1)	22.5 (21.2, 23.8)	24.5 (23.2, 26.0)	28.2 (26.9, 29.5)	1.57 (1.45, 1.69)
2006	24.1 (22.9, 25.4)	26.2 (24.8, 27.7)	27.9 (26.4, 29.4)	30.0 (28.5, 31.6)	34.3 (32.8, 35.8)	1.42 (1.33, 1.52)
2007	19.6 (18.6, 20.7)	20.2 (18.9, 21.4)	22.5 (21.2, 23.9)	24.3 (23.0, 25.7)	30.9 (29.6, 32.4)	1.58 (1.47, 1.69)
2008	22.5 (21.4, 23.6)	23.5 (22.2, 24.9)	25.9 (24.5, 27.3)	26.2 (24.8, 27.6)	31.5 (30.2, 32.9)	1.40 (1.31, 1.50)
2009	22.8 (21.6, 24.1)	26.7 (25.3, 28.2)	27.8 (26.3, 29.2)	31.8 (30.3, 33.3)	35.1 (33.7, 36.6)	1.54 (1.44, 1.65)
2010	23.3 (21.9, 24.7)	25.9 (24.5, 27.4)	25.2 (23.8, 26.6)	25.6 (24.3, 26.9)	30.0 (28.8, 31.3)	1.29 (1.20, 1.39)
2011	30.6 (28.9, 32.3)	30.4 (28.9, 31.9)	30.1 (28.6, 31.6)	29.5 (28.1, 31.0)	34.2 (32.8, 35.5)	1.12 (1.04, 1.20)
2012	26.3 (24.8, 27.9)	26.9 (25.5, 28.4)	28.6 (27.2, 30.1)	27.6 (26.3, 29.0)	32.3 (31.0, 33.6)	1.23 (1.14, 1.32)
2013	27.4 (25.9, 29.0)	26.4 (25.1, 27.9)	27.9 (26.6, 29.4)	28.1 (26.7, 29.5)	31.5 (30.2, 32.8)	1.15 (1.07, 1.23)
2014	32.8 (31.2, 34.4)	31.6 (30.1, 33.1)	32.5 (31.0, 34.1)	32.5 (31.1, 34.1)	37.2 (35.7, 38.6)	1.13 (1.06, 1.21)
2015	28.9 (27.5, 30.3)	31.4 (29.9, 32.9)	32.4 (30.9, 34.0)	32.5 (31.0, 34.1)	37.4 (35.9, 39.0)	1.30 (1.22, 1.38)
2016	30.1 (28.8, 31.5)	30.8 (29.3, 32.3)	32.8 (31.2, 34.4)	33.6 (32.0, 35.2)	39.1 (37.6, 40.8)	1.30 (1.22, 1.38)
2017	30.4 (29.0, 31.8)	29.2 (27.8, 30.7)	32.7 (31.2, 34.3)	35.7 (34.1, 37.4)	42.1 (40.5, 43.8)	1.39 (1.30, 1.47)
2018	30.2 (28.8, 31.6)	32.2 (30.7, 33.7)	32.2 (30.7, 33.8)	34.8 (33.2, 36.5)	39.7 (38.1, 41.3)	1.31 (1.24, 1.40)
2019	28.4 (27.1, 29.8)	34.3 (32.8, 35.9)	34.5 (32.9, 36.1)	35.7 34.1, 37.4)	39.4 (37.9, 41.1)	1.39 (1.30, 1.48)
2020	1.6 (1.3, 2.0)	2.0 (1.6, 2.4)	2.3 (1.9, 2.7)	2.1 (1.7, 2.5)	3.0 (2.6, 3.5)	1.92 (1.47, 2.50)
2021	20.3 (19.1, 21.5)	20.6 (19.5, 21.8)	21.2 (20.0, 22.4)	20.9 (19.6, 22.2)	22.0 (20.8, 23.2)	1.08 (1.00, 1.18)
All years	24.3 (24.0, 24.6)	25.5 (25.2, 25.8)	26.9 (26.6, 27.2)	28.0 (27.7, 28.4)	32.4 (32.1, 32.7)	
AAPC, % (95% CI)[b]	3.07 (1.83, 4.32)[c]	3.03 (1.92, 4.15)[c]	2.46 (1.63, 3.31)[c]	2.19 (1.21, 3.18)[c]	1.89 (1.00, 2.79)[c]	
% difference AAPC (95% CI)	ref	-0.04 (-1.57, 1.49)	-0.60 (-1.97, 0.77)	-0.88 (-2.33, 0.57)	-1.17 (-2.57, 0.22)	

[a]Data are expressed as No. (%). The percentage in each row were calculated using the total number as the denominator.

[b]Trends were quantified using version 5.0.2 of the Joinpoint Regression program (National Cancer Institute). No joinpoints were identified. AAPC was estimated for years 2004-2005 to 2019-2020.

The annual percentage change over the period 2004-2019 is identical to the average for both subgroups.

[c]The AAPC was significantly different from zero at $p<0.001$.

AAPC, Average Annual Percentage Change

© 2024 Mahant S et al. *JAMA Network Open.*

eTable 4. Trends in Bronchiolitis Hospitalization Rates Among Equity Stratifiers: Female vs. Male, 2004-2005 to 2021-2022

	**Total Person-Years, No.** 5,082,191						
	**Total Hospitalizations, No.** 58,215						
	**Male**			**Female**			
**Year**	**Hospitalization, No.**	**Person-Years, No.**	**Hospitalization, No. per 1000 person-years (95% CI)**	**Hospitalization, No.**	**Person-Years, No.**	**Hospitalization, No. per 1000 person-years (95% CI)**	**Rate Ratio = Female/Male**
2004	2,337	141,382	16.5 (15.9, 17.2)	1,519	133,860	11.4 (10.8, 11.9)	0.69 (0.64, 0.73)
2005	1,901	143,197	13.3 (12.7, 13.9)	1,189	135,216	8.8 (8.3, 9.3)	0.66 (0.62, 0.71)
2006	2,425	143,608	16.9 (16.2, 17.6)	1,543	135,939	11.4 (10.8, 11.9)	0.67 (0.63, 0.72)
2007	1,893	145,811	13.0 (12.4, 13.6)	1,254	138,464	9.1 (8.6, 9.6)	0.70 (0.65, 0.75)
2008	1,834	147,949	12.4 (11.8, 13.0)	1,166	140,800	8.3 (7.8, 8.8)	0.67 (0.62, 0.72)
2009	2,108	147,092	14.3 (13.7, 15.0)	1,428	139,652	10.2 (9.7, 10.8)	0.71 (0.67, 0.76)
2010	1,749	142,871	12.2 (11.7, 12.8)	1,145	135,467	8.5 (8.0, 9.0)	0.69 (0.64,0.74)
2011	2,205	143,827	15.3 (14.7, 16.0)	1,631	136,626	11.9 (11.4, 12.5)	0.78 (0.73, 0.83)
2012	2,053	146,762	14.0 (13.4, 14.6)	1,328	139,300	9.5 (9.0, 10.1)	0.68 (0.64, 0.73)
2013	1,928	146,235	13.2 (12.6, 13.8)	1,273	138,822	9.2 (8.7, 9.7)	0.70 (0.65, 0.75)
2014	2,287	144,928	15.8 (15.1, 16.4)	1,476	137,976	10.7 (10.2, 11.3)	0.68 (0.63, 0.72)
2015	2,030	145,037	14.0 (13.4, 14.6)	1,335	138,030	9.7 (9.2, 10.2)	0.69 (0.64, 0.74)
2016	2,030	145,436	14.0 (13.4, 14.6)	1,404	137,960	10.2 (9.7, 10.7)	0.73 (0.68, 0.78)
2017	2,154	145,514	14.8 (14.2, 15.4)	1,444	137,847	10.5 (9.9, 11.0)	0.71 (0.66, 0.76)
2018	2,192	145,471	15.1 (14.4, 15.7)	1,563	137,600	11.4 (10.8, 11.9)	0.75 (0.71, 0.81)
2019	2,275	145,436	15.6 (15.0, 16.3)	1,594	138,140	11.5 (11.0, 12.1)	0.74 (0.69, 0.79)
2020	133	143,853	0.9 (0.8, 1.1)	80	136,885	0.6 (0.5, 0.7)	0.63 (0.48, 0.84)
2021	1,351	143,115	9.4 (8.9, 10.0)	958	136,084	7.0 (6.6, 7.5)	0.75 (0.69, 0.81)
All years	34,885	2,607,523	13.4 (13.2, 13.5)	23,330	2,474,668	9.4 (9.3, 9.6)	
AAPC[b], % (95% CI)			0.22 (-0.94, 1.40)			0.75 (-0.60, 2.12)	
% difference AAPC (95% CI)			ref			0.53 (-1.11, 2.17)	

[a]Data are expressed as No. (%). The percentage in each row were calculated using the total number as the denominator.
[b]Trends were quantified using version 5.0.2 of the Joinpoint Regression program (National Cancer Institute). No joinpoints were identified. AAPC was estimated for years 2004-2005 to 2019-2020.
The annual percentage change over the period 2004-2019 is identical to the average for both subgroups.
AAPC, Average Annual Percentage Change

© 2024 Mahant S et al. *JAMA Network Open.*

eTable 5. Trends in Bronchiolitis Hospitalization Rates Among Equity Stratifiers: Rural vs. Urban, 2004-2005 to 2021-2022

	Total Person-Years, No. 5,082,191						
	Total Hospitalizations, No. 58,215						
	Urban			Rural			
Year	Hospitalization, No.	Person-Years, No.	Hospitalization, No. per 1000 person-years (95% CI)	Hospitalization, No.	Person-Years, No.	Hospitalization, No. per 1000 person-years (95% CI)	Rate Ratio = Rural/Urban
2004	3,331	244,519	13.6 (13.2, 14.1)	521	29,650	17.6 (16.1, 19.2)	1.29 (1.17, 1.42)
2005	2,738	249,101	11.0 (10.6, 11.4)	348	28,745	12.1 (10.9, 13.5)	1.10 (0.98, 1.23)
2006	3,391	250,862	13.5 (13.1, 14.0)	570	28,376	20.1 (18.5, 21.8)	1.49 (1.36, 1.63)
2007	2,794	255,248	11.0 (10.5, 11.4)	351	28,793	12.2 (11.0, 13.5)	1.11 (0.99, 1.25)
2008	2,617	259,200	10.1 (9.7, 10.5)	380	29,290	13.0 (11.7, 14.4)	1.28 (1.15, 1.43)
2009	3,049	257,312	11.9 (11.4, 12.3)	480	29,170	16.5 (15.0, 18.0)	1.39 (1.26, 1.53)
2010	2,541	249,751	10.2 (9.8, 10.6)	350	28,319	12.4 (11.1, 13.7)	1.21 (1.08, 1.36)
2011	3,349	251,633	13.3 (12.9, 13.8)	484	28,514	17.0 (15.5, 18.6)	1.28 (1.16, 1.41)
2012	2,970	256,872	11.6 (11.2, 12.0)	409	28,882	14.2 (12.8, 15.6)	1.22 (1.10, 1.36)
2013	2,864	255,915	11.2 (10.8, 11.6)	329	28,831	11.4 (10.2, 12.7)	1.02 (0.91, 1.15)
2014	3,335	254,057	13.1 (12.7, 13.6)	422	28,375	14.9 (13.5, 16.4)	1.13 (1.02, 1.26)
2015	2,987	254,733	11.7 (11.3, 12.2)	371	27,664	13.4 (12.1, 14.9)	1.14 (1.02, 1.28)
2016	3,022	255,348	11.8 (11.4, 12.3)	404	27,279	14.8 (13.4, 16.3)	1.25 (1.13, 1.39)
2017	3,174	255,027	12.5 (12.0, 12.9)	411	27,459	15.0 (13.6, 16.5)	1.20 (1.08, 1.34)
2018	3,307	254,236	13.0 (12.6, 13.5)	440	27,826	15.8 (14.4, 17.4)	1.22 (1,10, 1.35)
2019	3,431	254,791	13.5 (13.0, 13.9)	428	27,748	15.4 (14.0, 17.0)	1.15 (1.03, 1.27)
2020	178	252,696	0.7 (0.6, 0.8)	34	27,158	1.3 (0.9, 1.8)	1.78 (1.22, 2.58)
2021	2,051	250,998	8.2 (7.8, 8.5)	255	27,434	9.3 (8.2, 10.5)	1.14 (1.00, 1.30)
All years	51,129	4,562,301	11.2 (11.1, 11.3)	6,987	509,513	13.7 (13.4, 14.0)	
AAPC[b], % (95% CI)			0.52 (-0.65, 1.70)			-0.10 (-1.95, 1.79)	
% difference AAPC (95% CI)			ref			-0.62 (-2.63, 1.40)	

[a]Data are expressed as No. (%). The percentage in each row were calculated using the total number as the denominator.

[b]Trends were quantified using version 5.0.2 of the Joinpoint Regression program (National Cancer Institute). No joinpoints were identified. AAPC was estimated for years 2004-2005 to 2019-2020.

The annual percentage change over the period 2004-2019 is identical to the average for both subgroups.

AAPC, Average Annual Percentage Change

© 2024 Mahant S et al. *JAMA Network Open.*

eTable 6. Trends in Bronchiolitis Hospitalization Rates Among Equity Stratifiers: Material resources quintile, 2004-2005 to 2021-2022

	**Total Person-Years, No.** 5,082,191					
	**Total Hospitalizations, No.** 58,215					
	**Hospitalizations, No.** 9,823	**Hospitalizations, No.** 9,880	**Hospitalizations, No.** 10,291	**Hospitalizations, No.** 10,994	**Hospitalizations, No.** 15,757	
	**Q1**	**Q2**	**Q3**	**Q4**	**Q5**	
**Year**	**Hospitalization, No. per 1000 person-years (95% CI)**	**Hospitalization, No. per 1000 person-years (95% CI)**	**Hospitalization, No. per 1000 person-years (95% CI)**	**Hospitalization, No. per 1000 person-years (95% CI)**	**Hospitalization, No. per 1000 person-years (95% CI)**	**Rate Ratio = Q5/Q1**
2004	11.2 (10.4, 12.1)	12.4 (11.4, 13.4)	12.8 (11.8, 13.8)	14.6 (13.5, 15.7)	17.8 (16.8, 18.9)	1.59 (1.44, 1.75)
2005	8.8 (8.1, 9.6)	9.9 (9.0, 10.8)	10.5 (9.7, 11.5)	11.8 (10.8, 12.8)	13.8 (12.9, 14.8)	1.57 (1.41, 1.75)
2006	11.0 (10.2, 11.8)	12.7 (11.7, 13.7)	14.1 (13.1, 15.2)	14.3 (13.2, 15.4)	17.4 (16.3, 18.4)	1.58 (1.44, 1.74)
2007	9.3 (8.6, 10.0)	9.7 (8.8, 10.6)	10.5 (9.6, 11.4)	11.8 (10.9, 12.8)	13.9 (13.0, 14.8)	1.49 (1.35, 1.66)
2008	8.5 (7.8, 9.2)	9.4 (8.6, 10.3)	10.1 (9.3, 11.0)	10.4 (9.6, 11.4)	12.9 (12.0, 13.8)	1.52 (1.36, 1.69)
2009	9.5 (8.7, 10.3)	10.7 (9.9, 11.7)	11.2 (10.3, 12.2)	13.2 (12.3, 14.2)	15.5 (14.6, 16.5)	1.63 (1.47, 1.81)
2010	8.6 (7.8, 9.6)	9.5 (8.7, 10.4)	9.4 (8.6, 10.3)	10.9 (10.0, 11.8)	12.0 (11.3, 12.9)	1.39 (1.23, 1.57)
2011	13.4 (12.3, 14.6)	12.3 (11.3, 13.3)	13.4 (12.5, 14.5)	12.8 (11.9, 13.8)	15.3 (14.4, 16.2)	1.14 (1.03, 1.26)
2012	11.0 (10.0, 12.0)	11.2 (10.3, 12.1)	11.9 (11.0, 12.9)	11.0 (10.2, 11.9)	12.5 (11.7, 13.3)	1.14 (1.01, 1.27)
2013	11.2 (10.3, 12.3)	10.2 (9.4, 11.1)	11.1 (10.2, 12.0)	11.1 (10.2, 12.0)	11.9 (11.1, 12.7)	1.06 (0.95, 1.19)
2014	12.6 (11.6, 13.6)	11.9 (11.0, 12.9)	12.4 (11.5, 13.4)	13.1 (12.2, 14.1)	15.0 (14.1, 15.9)	1.19 (1.07, 1.32)
2015	10.5 (9.7, 11.4)	11.0 (10.1, 11.9)	11.6 (10.7, 12.6)	11.4 (10.5, 12.3)	14.1 (13.2, 15.0)	1.34 (1.21, 1.49)
2016	10.7 (9.9, 11.6)	11.0 (10.1, 11.9)	11.1 (10.2, 12.0)	12.4 (11.4, 13.4)	14.6 (13.7, 15.6)	1.36 (1.23, 1.51)
2017	10.7 (9.9, 11.5)	11.2 (10.3, 12.1)	11.6 (10.7, 12.6)	13.7 (12.7, 14.8)	15.9 (14.9, 16.9)	1.49 (1.34, 1.65)
2018	11.5 (10.7, 12.4)	12.6 (11.7, 13.6)	12.6 (11.6, 13.6)	13.5 (12.5, 14.5)	15.8 (14.8, 16.9)	1.37 (1.24, 1.52)
2019	10.5 (9.7, 11.4)	12.3 (11.4, 13.3)	14.5 (13.5, 15.5)	14.1 (13.1, 15.2)	16.2 (15.2, 17.2)	1.54 (1.39, 1.71)
2020	0.5 (0.3, 0.8)	0.6 (0.4, 0.8)	0.9 (0.7, 1.2)	0.6 (0.4, 0.8)	1.1 (0.8, 1.4)	2.11 (1.33, 3.33)
2021	7.3 (6.6, 8.1)	8.4 (7.7, 9.2)	7.6 (6.9, 8.3)	8.6 (7.8, 9.4)	9.4 (8.6, 10.2)	1.28 (1.12, 1.47)
All years	9.8 (9.6, 10.0)	10.3 (10.1, 10.5)	10.9 (10.7, 11.1)	11.6 (11.4, 11.8)	13.6 (13.4, 13.8)	
AAPC[b], % (95% CI)	1.02 (-0.44, 2.51)	0.66 (-0.53, 1.87)	0.64 (-0.76, 2.06)	-0.32 (-2.84, 2.27)	0.09 (-2.41, 2.65)	
% difference AAPC (95% CI)	ref	-0.36 (-2.10, 1.38)	-0.38 (-2.25, 1.48)	-1.34 (-4.23, 1.55)	-0.93 (-3.80, 1.93)	

[a]Data are expressed as No. (%). The percentage in each row were calculated using the total number as the denominator.

[b]Trends were quantified using version 5.0.2 of the Joinpoint Regression program (National Cancer Institute). AAPC was estimated for years 2004-2005 to 2019-2020. Joinpoint analysis showed no significant trends in hospitalization rate by material resources quintile. No joinpoints were identified for quintiles 1 through 3. In Quintile 4, there was an initial decline in hospitalization rate from 2004-2008 (Trend 1: APC=-5.72 (95%CI: -14.10, 3.49), p=0.19) followed by an incline from 2008-2019 (Trend 2: APC=1.72 (95%CI:-0.28, 3.76), p=0.09). In Quintile 5, there was an initial decline in hospitalization rate from 2004-2012 (Trend 1: APC=-3.05 (95%CI: -6.51, 0.53), p=0.09) followed by an incline from 2012-2019 (Trend 2: APC=3.81 (95%CI:-0.70, 8.53), p=09). However, these trends for quintiles 4 and 5 were not statistically significant. The Average Annual Percent Change (AAPC) for each of the 5 quintile subgroups was not significantly different from zero at the alpha = 0.05 level. The AAPC difference between quintiles 2 to 5 and referent quintile 1 were not significantly different zero at the alpha = 0.05 level.

© 2024 Mahant S et al. *JAMA Network Open.*

eAppendix. Canadian Paediatric Inpatient Research Network Members

First Name and Middle Initial(s)	Last Name	Academic Degrees	Institution	Location (city, state/province, country)	Role or Contribution
Gita	Wahi	MD	McMaster Children's Hospital, McMaster University	McMaster, Ontario, Canada	Research Lead
Lucy	Giglia	MD	McMaster Children's Hospital, McMaster University	McMaster, Ontario, Canada	Site Lead
Ann	Bayliss	MD	Trillium Health Partners; University of Toronto	Mississauga, Ontario, Canada	Site Lead
Mahmoud	Sakran	MD	Lakeridge Health; Queens University	Oshawa, Ontario, Canada	Community Hospital Lead
Ronik	Kanani	MD	North York General Hospital; University of Toronto	Toronto, Onario, Canada	Site Lead
Anupam	Seghal	MB DNB	Queen's University	Kingston, Ontario, Canada	Site Lead
Melanie	Buba	MD	Children's Hospital of Eastern Ontario; University of Ottawa	Ottawa, Ontario, Canada	Site Lead
Sean	Murray	MD	Health Sciences North; Northern Ontario School of Medicine	Sudbury, Ontario, Canada	Site Lead
Sepideh	Taheri	MBChB	Children's Hospital London Health Sciences Centre; Western University	London, Ontario, Canada	Site Lead

© 2024 Mahant S et al. *JAMA Network Open.*
