# Supplementary material for: Sociodemographic Factors and Trends in Bronchiolitis-Related Emergency Department Visit and Hospitalization Rates
Source: JAMA Netw Open. 2024 Apr 29;7(4):e248976. doi: 10.1001/jamanetworkopen.2024.8976 (PMC11059049; doi:10.1001/jamanetworkopen.2024.8976)
Supplement: Supplement 2. — Data Sharing Statement [file jamanetwopen-e248976-s002.pdf]

Data Sharing Statement

Mahant. Sociodemographic Factors and Trends in Bronchiolitis-Related Emergency Department Visit and Hospitalization Rates. *JAMA Netw Open*. Published May 01, 2024. doi:10.1001/jamanetworkopen.2024.8976

Data
Data available: No
